# “I wish it had a place to go”: a nominal group study of barriers to the effectiveness of non-surgical treatments for knee osteoarthritis inclusive of minority populations

**DOI:** 10.1186/s13075-021-02676-8

**Published:** 2021-12-01

**Authors:** Jasvinder A. Singh

**Affiliations:** 1 Medicine Service, Birmingham VA Medical Center, Birmingham, AL USA; 2 Department of Medicine at School of Medicine, Division of Epidemiology at School of Public Health, University of Alabama, Faculty Office Tower 805B, 510 20th Street S, Birmingham, AL 35294 USA

**Keywords:** Knee osteoarthritis, Barriers, Qualitative, Nominal group, NGT, Race, African Americans, Weight loss, Exercise, Medication, Effectiveness

## Abstract

**Objective:**

To examine patient experience, views, and opinions regarding the ineffectiveness of the current knee osteoarthritis (OA) treatments.

**Methods:**

Nominal groups were conducted with consecutive clinic patients with knee OA, oversampling African Americans. Patients discussed and rank-ordered their concerns.

**Results:**

Fourteen nominal groups with 48 knee OA patients were conducted with a mean age of 60.6 years (standard deviation, 9.8) and a knee OA duration of 7.8 years (sd, 5.4); 25% were men, and 54% were African American. The most frequently cited highly ranked concerns for the ineffectiveness of current knee OA treatments were as follows: (1) medication-related—(A) side effects (3 groups; 4% vote), (B) limited efficacy (5 groups; 11% vote), (C) medication not targeting underlying disease (7 groups; 16% vote), (D) lack of personalized medication use (3 groups; 4% vote), (E) temporary benefit (3 groups; 6% vote), and (F) fear of addiction/natural treatment preference (2 groups; 3% vote); (2) exercise/physical therapy-related—(G) exacerbation of joint pain (1 group; 3% vote), (H) difficulty in doing exercises (2 groups; 2% vote), (I) lack of motivation (8 groups; 12% vote), (J) technical challenges/lack of personalized exercise regimens (1 group; 1% vote), and (K) cost (2 groups; 3% vote); and (3) weight loss-related—(L) difficulty in achieving weight loss (4 groups; 6% vote) and (M) motivation (1 group; 1% vote).

**Conclusions:**

A representative sample of participants with knee OA identified several barriers to the effectiveness of current knee OA treatments. This new knowledge provides insights for making the current treatment options potentially more usable and/or more effective.

**Supplementary Information:**

The online version contains supplementary material available at 10.1186/s13075-021-02676-8.

## Key messages

1. Participants with knee osteoarthritis (OA) experienced several limitations of the current treatments, including those to the effective use of medications, physical therapy, exercise, and weight loss.
2. The main barriers to the effectiveness of medications for treating knee OA symptoms were medication side effects, limited efficacy, temporary relief, the lack of personalized approach, and medication not targeting the underlying disease, i.e., cartilage repair.
3. Exacerbation of pain, difficulty continuing regimen, lack of motivation, technical aspects, and cost were barriers to the effectiveness of exercise/physical therapy as a treatment for knee OA.
4. Difficulty in achieving weight loss, the lack of motivation, and perceived limited efficacy were the main barriers to the effectiveness of weight loss as a knee OA treatment.

## Background

Osteoarthritis (OA) is a disabling chronic disease that affects 10% of adult Americans [1, 2]. OA is the most common type of arthritis in adults. In the USA, $185.5 billion in annual insurer expenditures are attributable to medical care for patients with OA [3]. Women with OA had $4833 greater annual insurer expenditures and an additional $1379 in out-of-pocket costs compared with women without OA [3]. Globally, of the 291 conditions, hip and knee OA was ranked as the 11th highest contributor to global disability in the Global Burden of Disease Study [4]. The USA currently has the highest age-standardized prevalence of OA in the world [5]. Symptomatic knee OA affects 17% of Americans 45 years or older [2]. Knee OA is one of the most common causes of lower extremity disability [6] with an estimated lifetime risk of 40% in men and 47% in women [7]. Knee OA is associated with significantly worse quality of life and function and higher rates of disability [8–12]. The public health impact of knee OA continues to rise with an aging population in the USA [13]. Compared to Caucasians, African Americans have a similar prevalence of knee OA [14, 15] and worse pain and function [16, 17]. Thus, knee OA is a major public health problem, especially in racial/ethnic minorities.

Many treatment options, pharmacological and non-pharmacological, are available for people with knee OA [18]. The American College of Rheumatology (ACR) and Arthritis Foundation (AF) recently published for the management of knee OA. Strong recommendations from knee OA treatment guidelines (i.e., those that should be applied to the vast majority of patients because there is a high level of certainty that the benefits outweigh the harms) include oral and topical NSAIDs, intra-articular corticosteroids, exercise, weight loss (when appropriate), tai chi, self-management, and tibiofemoral braces [18]. While the use of non-steroidal anti-inflammatory drugs (NSAIDs) is widespread, non-pharmacologic modalities are grossly underutilized by people with knee OA [19]. A large proportion of patients with knee OA are not satisfied with current treatments [20].

A recent systematic review of qualitative studies in knee OA that included 21 studies with > 600 patients confirmed the impact of OA on patient’s daily activities, social participation, and emotional health in knee OA [21]. To date, most qualitative research in knee OA has focused on examining knowledge related to OA, patterns of treatment, and barriers to treatment adherence or physician perspective, views, and experience. To our knowledge, only limited insight is available from the qualitative studies focused on the patient perspective of why these currently available effective treatments are not being used frequently and/or effectively by patients with knee OA. Therefore, the objective of this study was to examine patient experience, views, and opinions and reasons for why the current knee OA treatments were not effective for people with knee OA. In this study, African Americans and women were oversampled to get a diverse patient perspective.

## Methods

### Study sample

Consecutive patients at a community-based outpatient clinic affiliated with University of Alabama at Birmingham (UAB), Birmingham, AL, with at least one outpatient visit with an International Classification of Diseases, tenth revision, common modification (ICD-10-CM) code M17.x for knee OA between January 2019 and August 2020, were invited to participate. African Americans were oversampled in this study due to their under-representation in qualitative research in knee OA. Study participants received a $30 check for participation. The UAB Institutional Review Board (IRB) approved the study.

### Nominal group technique (NGT) sessions and analyses

Patient nominal group sessions lasted 1.5–2 h each between July and November 2020. The study PI (J.S.) drafted the study question with two potential formulations and shared it with patients with knee osteoarthritis at the UAB rheumatology clinic. The question was also reviewed separately with other providers and researchers. An iterative process that included several discussions with various stakeholders led to the final study question: “Why do you think current treatments (medicines, physical therapy, exercise, weight loss) for osteoarthritis (arthritis due to loss of cartilage; wear and tear arthritis) of the knee joint do not work?”

The study PI (J.A.S), with an extensive experience in conducting nominal groups [22, 23], led the NGT sessions. The NGT is similar to the traditional focus group in that it taps the experiences, skills, or feelings of the participants, but in contrast to the focus group, promotes an even participation. The NGT has been used successfully in a variety of medical conditions [24–30]. A focus group is well suited to understand the breadth of an issue, while a nominal group is well suited for an in-depth examination of a question. The NGT methods lead to the development of an inclusive list of issues related to a specific question followed by soliciting feedback on the relative importance of this list using a rank-ordering procedure [27, 31]. For patient safety during the COVID-19 pandemic, these NGTs were conducted virtually using HIPAA-compliant zoom sessions [32], instead of the traditional in-person session. All participants provided informed consent prior to the initiation of the discussion [32].

Each session started with introductions by participants, including their age and duration of knee OA and its impact on their lives. Before starting the NGT meeting, the moderator asked the participants if the question was clear; all questions/clarifications were addressed prior to starting the NGT session. The moderator showed the main question on the screen, which was also read out aloud slowly by the moderator, so that each participant could write it on the top of a blank sheet of paper. The moderator asked each participant to confirm that they had had experience with at least one or more of the knee OA treatment options being discussed, i.e., medication, physical therapy, exercise, and weight loss. All participants confirmed this prior to the nominal group discussion.

Next, the NGT participants briefly and independently generated as many words or short phrases as possible in response to the question on a sheet of paper, without any discussion with each other. Participants then nominated a single response each in a round-robin fashion, which were recorded verbatim by the NGT moderator (J.A.S.) on a power-point slide in large letters so that it was visible to each participant on the zoom screen, whether they were using a phone, touchpad computer, or a desktop computer. These responses were nominated and collected until all responses identified by any participant were recorded. This step was completed without any comments from other participants or the moderator. Once a complete list of all nominated responses was complied, participants discussed and elaborated on each response and provided their perspectives, and where appropriate combined responses that were very similar. Finally, each participant identified and scored the top three responses among the nominated responses deemed most important for people with knee OA from 1 to 3 on a piece of paper, 3 being the highest score. After each participant had completed their rank score, they read out their nominated response and the rank to the moderator, one participant at a time. A rank order was created for nominated responses based on total scores for each nominal group, with the highest score corresponding to the top rank.

High-rank ordering of responses across the nominal groups was analyzed. Responses were compared to determine the overlap and ensure saturation, as the nominal group sessions were completed. Nominal groups were conducted until significant overlap was noted, and saturation was confirmed. Each NGT was audio recorded. An administrative assistant (D.F.) fully transcribed the NGT discussions. Transcriptions were examined to confirm the accuracy of all nominated responses and examine all statements made relative to each response (discussions directly connected, solutions generated, etc.), which led to the creation of a comprehensive list of statements.

## Results

### Study participant characteristics

Of the 73 eligible consecutive people, 16 could not be reached on the phone, 4 reported that they did not have a diagnosis of knee osteoarthritis, and 5 were not interested in participating. Fourteen nominal groups with 48 patients (2–8/group; see Additional file 1 for details) with knee OA were conducted. The mean was 60.6 years (standard deviation, 9.8), 75% were women and 54% were African American, and the mean knee OA disease duration was 7.8 years. The diagnosis of knee OA was confirmed for each participant by self-report of a physician diagnosis of knee OA before starting the NGT (in addition to ICD-10-CM for knee OA in medical records; Table 1). Of the responders to baseline electronic survey, 33% had a college or associate degree, 24% were married, and 19% were retired (Table 1). Of these, 35% were on oral analgesics, 24% were using NSAIDs, 16% were on opioid analgesics, 27% were using topical creams/gels, and 16% each were using intra-articular glucocorticoids or hyaluronic acid injections. Each participant had had an experience with at least one or more of the knee OA treatment options under discussion, i.e., medication, physical therapy, exercise, and weight loss. All participants were aware of each modality, even if they had not tried each of that these modalities for treating their knee OA. The saturation of themes was achieved.

**Table 1:** Demographics of nominal group participants of people with knee osteoarthritis (*n* = 48)

	***N*** (%), unless otherwise specified
**Characteristics reported during the nominal group sessions**	
Age in years, mean (*SD*)	60.6 (9.8)
Male sex, *N* (%)	12 (25%)
Race/ethnicity, *N* (%)	
White	22 (46%)
African American	26 (54%)
Knee osteoarthritis disease duration in years, mean (*SD*)	7.8 (5.4)
**Additional characteristics reported in an online patient survey**^a^	
Education, *N* (%)	
High school graduate	6 (16%)
Some college, no degree	1 (3%)
Trade, technical, or vocational training	2 (5%)
Associate degree	6 (16%)
Bachelor’s degree	5 (14%)
Master’s degree	1 (3%)
Missing	16 (43%)
Marital status, *N* (%)	
Single	3 (8%)
Married	9 (24%)
Widowed	2 (5%)
Divorced	7 (19%)
Missing	16 (43%)
Current employment, *N* (%)	
Employed	1 (3%)
Homemaker	1 (3%)
Student	1 (3%)
Retired	7 (19%)
Unable to work	10 (27%)
Missing	17 (46%)
Other joints with osteoarthritis, *N* (%)	
Hip	7 (19%)
Hand	8 (22%)
Back	7 (19%)
Neck	3 (8%)
Missing	12 (32%)
Joints replaced, *N* (%)	
One knee	3 (8%)
Both knees	3 (8%)
Current treatments including medications^b^, *N* (%)	
Oral analgesics including acetaminophen	13 (35%)
Non-steroidal anti-inflammatory drugs (NSAIDs)	9 (24%)
Opioid analgesics	6 (16%)
Oral glucocorticoids	3 (8%)
Local applications including Voltaren, capsaicin, or other creams	10 (27%)
Intraarticular glucocorticoid injection	6 (16%)
Other intraarticular injections including hyaluronic acid	6 (16%)
Muscle-strengthening, including exercise or physical therapy	4 (11%)
Weight loss	5 (14%)
Tai chi	1 (3%)
KOOS subscale scores, mean (*SD*), range 0–100 mm	
Pain	53.3 (47.2)
Symptoms	49.3 (21.6)
Activities of daily living (ADL)	55.5 (19.9)
Sport/recreation	34.2 (31.0)
Quality of life (QOL)	23.8 (22.5)

Age, sex, race/ethnicity, and OA disease duration were reported during the nominal group by each patient, and therefore, these data were available for all participant

*KOOS* Knee Osteoarthritis And Outcomes Survey, a validated measure of outcomes in knee osteoarthritis, with 100 indicating better outcome

^a^The source of these data was an online patient survey, which was only completed by 37 of the 48 people which is the denominator for these data, many of whom did not answer all the questions

^b^The total adds up to more than 37, since many participants used more than one treatment modality

### Participant responses from nominal groups

The top nominated responses from each nominal group are listed in Table 2 (see Additional file 1 for details). In the section below, the top 3 responses nominated by at least one nominal group are listed.

**Table 2:** Main themes and their ranking by each respective nominal group for barriers to current treatments not working for knee osteoarthritis (OA)

Why do you think current treatments (medicines, therapy, weight loss, exercise) for osteoarthritis (arthritis due to loss of cartilage; wear and tear arthritis) of the knee joint do not work?	Score
**NGT1: Q1: 2 people: 2 females: 1 White, 1 African American (12 votes)**	
**B.** The weight loss would help, but it’s not easy.	6
**D.** My insurance doesn’t allow me to do therapy as long as I would like to do it.	3
**G.** Pills have side effects..	3
***A.*** *Shots in my knees, have to be done repeatedly.*	*0*
***C.*** *Need to do physical therapy for a long time, 6 weeks at a time, it’s very hard.*	*0*
***E.*** *Pandemic caused a lot of problems, you can’t have anything done- getting physical therapy.*	*0*
***F.*** *Most of the medicines don’t work. It’s temporary; the pain comes back.*	*0*
**NGT2: 4 people: 4 females: 1 White, 3 African Americans (24 votes)**	
**D**. Medicine helps some, but pain persists.	7
**E.** Medicine has side effects.	4
**C.** I got an injection that relieved my pain, and it lasted only 3 months.	4
**F.** Weight loss is difficult.	3
**B.** Exercise makes knee pain worse.	2
**G.** Therapy: I am unable to do it, as I should.	2
**H.** Therapy making OA symptoms worse.	2
***A.*** *Problem doing exercise.*	*0*
**NGT3: 3 people: 1 male, 2 females: 1 White, 2 African Americans (18 votes)**	
**C.** Pain remedies only address pain: treatments are only prophylactic, and only for the most difficult days.	5
**E.** Sometimes we have more weight that causes us to have more pain.	4
**B.** The missing cartilage is still missing. Activity and weather still affect the pain. Until something is done for the cartilage, the pain will continue.	3
**F.** We don’t exercise the way we should exercise.	3
**A.** I feel like it’s not working, because I am not taking the right medicine. The pain is still there. I feel like my medicine should be changed.	2
**G.** The weather—cold weather.	1
***D.*** *In order to function, I have pain and swelling, so there is some trade-off.*	*0*
**NGT4: 8 people: 1 male, 7 females: 4 White, 4 African Americans (48 votes)**	
**A.** After your cartilage is gone, it’s just bone on bone, I don’t think any treatment other than the surgery will work.	21
**B.** According to me, it’s just trial and error, some meds may work for some people, and some don’t; they don’t last a life time; I have taken medicine and I have had to go up my medicine, because half the time it doesn’t work and when it does work it does not last long.	8
**C.** Medicine side effects to certain meds.	4
**G.** I am not able to exercise because of pain/weak muscles.	4
**D.** Being too overweight, the exercise can exacerbate the wear.	3
**F.** I have had several injections that don’t work/need them more frequently.	3
**H.** If the medication doesn’t work within the first couple of days, a lot of people will quit taking it, because they don’t see the benefit..	3
**I.** You are not following the proper medicine regimen.	2
***E.*** *Some medicines make the wear of the joint quicker.*	*0*
**NGT5: 4 people: 2 males; 2 females: 2 White; 2 African Americans (24 votes)**	
**A.** Medicine: the reason I think it doesn’t work very well, because I take other medications. And I have to take this medication to counteract the other medication, so that It won’t affect my liver or my kidney. That’s one of my opinion is why I think the mediation doesn’t work.	7
**B.** Weight loss: MOTIVATION: I feel sometimes it is easy to get started losing weight. In the second week, it’s hard to move around and get tired and have to slow down. And getting depressed and getting something to eat, and weight loss, sometimes, you want to give up.	4
**L.** By the time you start you start any treatment, it’s too late.	3
**C.** Weight loss: difficulty getting adequate weight loss.	2
**D.** Physical therapy: TOO MUCH, cause too much pain: which I experienced. I went to the therapy; it all depends on who you have. Some therapists don’t push you enough and some push you too hard. “You can do few more and sometimes it does more damage.”	2
**G.** Exercises: lack of equipment.	2
**J.** Injections: incorrect spot: first of all, the injections, I have had many. Most of them don’t work. Sometimes I think injections are not in the right place.	2
**K.** Overall, the problem is hereditary. And nothing would work.	1
**E.** Physical therapy: variable, varies by who you go to in PT.	1
***F.*** *Exercises: not knowing: while I do feel like I am ok when joints loosen up, I feel like I am 17 and then go out there and overdo it.*	*0*
***I.*** *Exercises: temporary relief.*	*0*
***H.*** *Exercises worsen pain: again, the exercise. I have done exercises, but if I overextend, it makes it worse.*	*0*
**NGT6: 4 people: 2 male, 2 females: 2 White, 2 African Americans (24 votes)**	
**A**. I believe that the treatments work on a limited basis to treat the symptoms.	8
**C**. Motivation/pain: it’s hard to get motivated when you are in pain.	8
**D**. However, once you lose the cartilage or it is damaged, then these treatments do not do anything to regenerate the cartilage.	5
**G**. I also have lupus and they say that’s part of my problem too—trouble with taking knee arthritis medications due to lupus.	1
**H**. My arthritis gets worse with daily activities, because I am active, and that’s why meds don’t work well for me.	1
**I**. My doctor has progressively gotten more aggressive with my meds based on my daily activities and my pain level; I don’t think that’s getting it.	1
***E****. Like for me, it’s hard to lose weight to be able to take off some pressure off my joints.*	*0*
***F****. Medical profession should look at other alternatives, rather than man-made medicines, like natural medicines.*	*0*
***B****. Motivation/time: when you get back to the real life, you can’t put so much work into, then when you are off.*	*0*
**NGT7: 4 people: 4 females: 2 White, 2 African Americans (24 votes)**	
**C.** Routine: exercise at least 15–20 min a day.	5
**H.** Lack of motivation/it takes commitment.	5
**D.** Weight loss may not work sometimes: watch your weight	3
**E.** Personalized treatment with medicines: because medicines haven’t been tested enough.	3
**A.** May be because I am not on the right diet—tomato, pasta.	3
**G.** It doesn’t build your cartilage.	3
**B.** Prepare your own food instead of eating out all the time.	1
**F.** Personalized therapy, weight loss, and exercise plan	1
***I.*** *Physical therapy can make you hurt*	*0*
**NGT8: 3 people: 3 females: 3 African Americans (18 votes)**	
**H.** I think there is a lot of natural remedies that can help rebuild without all of the medications that told us to take.	6
**B.** It’s the general medication or regimen for everybody; like you are doing one pill for everybody.	3
**C.** Individualized PT and exercise.	3
**A.** Pain interferes with the ability to do therapy—no immediate relief with PT or meds.	2
**E.** The damage is already done; there is nothing to help you healing of your knee, you have no cartilage.	2
**F.** Side effects with medication.	1
**G.** Injections only last 2–3 months.	1
***D.*** *With exercising with medication together might give them a sense of relief.*	*0*
**NGT9: 4 people: 1 male, 3 females: 1 White, 3 African Americans (24 votes)**	
**I.** Strain of physical work is interfering with treatment effectiveness.	10
**E.** Bone on bone cartilage is gone, treatments don’t work.	9
**A.** Medication: the reason why medicine doesn’t work, you have to keep constantly taking and there is a price point.	3
**C.** Paying for therapy, it does work, but you have constantly pay for that too.	1
**D.** Weight loss may have some effect on pain, but pain is still there.	1
***B.*** *No individualized medication treatment.*	*0*
***F.*** *No individualization of the strength training in your knees.*	*0*
***G.*** *Natural medicines, I believe, help; I believe vitamin D from Sun can also help.*	*0*
***H.*** *No plan how to go from physical therapy to exercises at home.*	*0*
***J.*** *Use hot and cold pack to improve effectiveness of these treatments.*	*0*
**NGT10: 2 people: 2 males: 3 White (12 votes)**	
**E.** Weight loss is difficult to get and to maintain.	5
**F.** Exercise may not work sometimes—a lot of people can’t do what I do- Motivation issues.	3
**A.** No cartilage left in my knees (and hips).	3
**C.** The pain; I had trouble with therapy and exercise with the pain.	1
***B.*** *Prescribed medicines don’t work: I don’t think the medicines worked and did what they were supposed to do.*	*0*
***D.*** *I wasn’t making any progress after some time with therapy, I thought I could make more progress myself.*	*0*
***G.*** *Taking better care of yourself and your life.*	*0*
**NGT11: 2 people: 1 male, 1 female: 2 White (12 votes)**	
**G.** Cost/access to exercise and weight loss programs.	6
**L.** Psychology/education for exercise: you have tried it before and sometimes you don’t know how to change your behavior.	4
**H.** Exercise: this comes down to time availability.	2
***A.*** *Weight loss: its daunting if you struggled with weight through your life, it could seem unachievable.*	*0*
***B.*** *Weight loss: lack of time to dedicate to weight loss.*	*0*
***C.*** *Weight loss: food prep.*	*0*
***D.*** *Weight loss: mental health aspects, depression, and body image issues.*	*0*
***E.*** *Confusion over contradicting diet plans.*	*0*
***F.*** *There is shor- term FU through medicine, and there is lack of modification effort—what was originally recommended doesn’t necessarily work.*	*0*
***I.*** *Exercise: pain especially and fatigue.*	*0*
***J.*** *Exercise: COVID, restricted access to weight training and other work-out facilities.*	*0*
***K.*** *Exercise: lack of motivation.*	*0*
**NGT12: 2 people: 2 females: 1 White, 1 African American (12 votes)**	
**C.** Motivation for weight loss and exercise: exercise is very hard to do because of what you need to do exercising, due to difficulty in exercising due to the pain.	3
**H.** Weight loss: challenges.	3
**A.** Medicine: can be addicting, and unable to take for long periods.	2
**B.** Medicines are usually only usually providing temporary relief of the pain.	2
**F.** Side effects of medicines.	1
**G.** Exercise: motivation due to pain in movement.	1
***D.*** *Physical therapy: strengthens muscles but doesn’t help with pain.*	*0*
***E.*** *Injections: short-term and painful.*	*0*
***I.*** *Most of these are short-term solutions.*	*0*
**NGT13: 2 people: 1 male, 1 female: 2 White (12 votes)**	
**A.** Medicine: help a little bit, but the problem is that it doesn’t happen quickly enough, the different things I take.	6
**E.** Lack of motivation for exercise.	4
**H.** Damage is done, even with weight loss and therapy.	2
***B.*** *The weight loss part is a big one for me, but can’t do.*	*0*
***C.*** *I can’t do exercises because of the knees.*	*0*
***D.*** *Talking therapy: pool, water therapy is good, but now, they have been shut down due to the COVID.*	*0*
***F.*** *Brace, which I have works, but I need help to put it on, but I have nobody here to help me to put it on.*	*0*
***G.*** *Creams are not strong enough for pain.*	*0*
***I.*** *Pool are good, but they are expensive, and the insurance does not cover it.*	*0*
**NGT14: 4 people: 2 males, 2 females: 2 White, 2 African Americans (24 votes)**	
**A.** Individual difference in response to Pills and creams: each individual is different, what might work for me, doesn’t work for another person.	6
**F.** Weight loss and exercise: no instant gratification, as human beings a lot of times we don’t want to finish what we start if no gratification.	4
**G.** Individual differences in disease: it strikes me as an important component of the individuals with pre-existing conditions, genetic predisposition to osteoarthritis.	4
**D.** Will power to continue the weight loss: don’t have the will power to keep going.	3
**J.** Difficulty in getting opioid Pain meds: Very difficult to get anything for actual pain, other than the anti-inflammatory agents.	3
**H.** Pain with the physical therapy: did not seem to be beneficial for the length of time, I did go. I didn’t seem to feel that I was benefiting much from it, other than the pain from the exercises.	2
**K.** Medicine works temporarily, because I feel that it numbs the information in your brain to stop hurting. Puts you in melatonin state to not feel the aching and hurting.	2
***B.*** *You don’t complete the treatment. Once you feel better, you stop using the cream or stop using the pills.*	*0*
***C.*** *Adherence to pills: not consistent, not taking the meds the same time every day.*	*0*
***E.*** *Become lazy with exercise and just give up.*	*0*
***I.*** *Problem taking medications due to other diseases: I found that most of the medications I tried had limitations. For example, taking the anti-inflammatory agents, because of my liver and kidney damage, they were not advised for long-term use.*	*0*
***L.*** *You don’t incorporate the therapy into lifestyle, once you stop the therapy.*	*0*

Each patient voted 3 points to the highest rank, 2 points to the second highest rank, and 1 point to the third highest rank. In most cases, the number of total votes is the number of patients × 6; Nominated responses are rearranged in this table based on votes from the highest to the lowest for ease of interpretation for the reader. This is also the reason for non-sequential letters for the responses

Nominated responses that did not receive votes after discussion are in italicized font

### Medication-related

#### Medication side effects and contraindications

Three of the 14 groups listed this among their top concerns, and six ranked this concern. It received 4% of all votes (11/288). Participants expressed concerns regarding the following: (1) side effects experienced, including gastrointestinal and renal side effects with NSAIDs; liver/hepatic side effects with acetaminophen; nausea, fatigue, skin rash, and headache; (2) medication contraindications such as renal failure and heart failure; and (3) worry about long-term potential harm to major body organ systems, including the kidneys, heart, and gastrointestinal system. A participant each from NGT1 and NGT2 said “I want to be alert during the day, if the medicine makes me groggy, I don’t want it. Ibuprofen makes me groggy” and “The majority of the medicines, anti-inflammatory medicines, I can’t take it at all, due to my stomach. Not over the counter not the prescribed one.”

#### Medicine has limited efficacy

Five of the 14 groups listed this among their top concerns, and nine ranked this concern. It received 11% of all votes (31/288). Several aspects were cited, including the following: (1) “trial and error” aspect regarding their use, (2) only a partial relief of pain, (3) short duration of pain relief and improvement in OA symptoms, (4) drug-drug interactions interfering with drug efficacy, and (5) the unpredictability of the relief duration with joint injections. Two participants from NGT1 said “Medicine helps some, but not much, I am constantly in pain” and “Trying to keep the pain down, but they don’t seem to help. So, what else to do to alleviate the pain”. Participants said “…half the time it doesn’t work and when it does work, it does not last long” (NGT3) and “I took a series of injections that didn’t work for me” (NGT2).

#### Medicine not targeting the underlying cause of knee OA

Seven of the 14 groups listed this among their top concerns, and seven ranked this concern. It received 16% of all votes (46/288). Participants were aware that most medications currently used for knee OA targeted joint pain, rather than the loss of cartilage, the underlying cause of knee OA. A participant from NGT3 said, “The missing cartilage is still missing. Activity and weather still affect the pain. Until something is done for the cartilage, the pain will continue.” Two participants from NGT4 commented “After your cartilage is gone, it’s just bone on bone, I don’t think any treatment other than the surgery will work” and “Because of the loss of cartilage, you have bone-on-bone and that causes the pain. Medicines do not work because they don’t duplicate the structure of the joint. With being bone-on-bone nothing to absorb the shock, so can’t recreate it.”

#### Medicine regimens not personalized

Three of the 14 groups listed this among their top concerns, and five ranked this concern. It received 4% of all votes (13/288). A participant said, “There are so many different medications that are available, what might work for one may not work for another person.” (NGT7), and “It’s the general medication or regimen for everybody; like you are doing one pill for everybody” (NGT8).

#### Medicine effect temporary

Three of the 14 groups listed this among their top concerns, and three ranked this concern. It received 6% of all votes (17/288). Participants were concerned about the need to have joint injections every 2–4 months due to the short-term efficacy of joint injections for pain relief. Participants said, “I got an injection that relieved my pain, and it lasted only 3 months” (NGT2); “I don’t know. The treatments are the treatments. They don’t make it go away, they make it better” (NGT6); and “Until we address the cause of the pain, treatments are not the cure” (NGT3).

#### Fear of medication addiction/natural treatments

One group each listed these among their top concerns. It received 3% of all votes (8/288). Participants were concerned about addiction to the prescription pain medications. They also preferred natural treatments to avoid harmful medication side effects. “I think there is a lot of natural remedies that can help rebuild without all of the medications that told us to take” (NGT8).

### Difficulty with exercise therapy

#### Exercise exacerbates knee joint pain

Two of the 14 groups listed this among their top concerns, and eight ranked this concern. It received 3% of all votes (10/288). Participants felt the exercise may exacerbate knee joint pain depending on the following: (1) the aggressiveness of the physical therapist, (2) their knee muscle strength and pain severity, (3) amount and type of exercises, (4) the past failure of exercises to improve symptoms, (5) the lack of right equipment or right instructions on how to do the exercises, and (6) the presence/absence of empathy in the physical therapist. Participants said “I feel like certain exercises may actually exacerbate the pain. So, I am not real sure, exactly what I should do. I swim every day for 30–45 min, and this includes bending my knees, which I believe increases the pain. But I am moving my entire body and feel this must help keep me strong. My knees hurt when I get back from swimming” (NGT2); “I went to the therapy; it all depends on who you have. Some therapists don’t push you enough and some push you too hard” (NGT5); “I had 2 types of therapists. One trying to increase my strength, that helped me a little, I had more pain related to those exercises. When I had pain, I didn’t keep it up. Another therapist’s aim was to help me stretch more and help me walk more optimally. That was very effective. It was little painful, but not serious. It just worked better for me” (NGT5); “My legs aren’t strong enough for me to exercise. I was a cheer leader, and did gymnastics, and now I don’t have the strength to exercise. I couldn’t even swim from one end to the other end because my legs were so weak” (NGT4); and “I feel like some care, some don’t. Some there to just make the hours and some really there to help someone” (NGT5).

#### Difficulty doing exercises

Two of the 14 groups listed this among their top concerns, and six ranked this concern. It received 2% of all votes (7/288). Participants had difficulty with the duration and specific types of exercises. Participants said “I do exercises at home. I can do hand and foot bike for 30 min” (NGT2); “I can’t do bending over due to pain” (NGT2); “Sometimes exercise causes more pain, and that’s why I don’t exercise” (NGT2); “Sitting in a chair and doing the stretches, we can do that. Easier than a bike or running” (NGT2); and “If you are moving too quickly or too much and the cartilage is missing, you are really causing inflammation when you are exercising, at least on the temporary basis. May be the exercise should be more directed to range of motion, rather than enough exercise to lose weight?” (NGT2).

#### Motivation to start and continue exercises

Eight of the 14 groups listed this among their top concerns, and eight ranked this concern. It received 12% of all votes (35/288). People had difficulty with several aspects of staying motivated due to the following: (1) the lack of a quick benefit; (2) slight worsening of knee pain initially, when starting exercises; (3) the time commitment; (4) remembering it; (5) not having a friend to do exercise with; and (6) exercise requires a change in behavior. Participants said, “You have to commit to PT; to exercises; to special diet; to our treatment plan- if you don’t commit it will not work” (NGT7); “I need motivation sometimes” (NGT7); “Exercise may not work sometimes – a lot of people can’t do what I do- Motivation” (NGT10); and “I struggle with it a lot; I have a lot of demands and stressed because I am a grad student, have to walk at night just to get the exercise” (NGT11).

#### Technical difficulty with exercises and/or the lack of a personalized exercise regimen

One of the 14 groups listed this among their top concerns, and one ranked this concern. It received 1% of all votes (3/288). Participants from NGT8 commented that “Everyone’s body make-up is not the same, so this will help”; “Just in general, the exercises they want you to do, it’s the same plan they are giving to everyone”; and “If you had an individual program prescribed for your bodyweight and body size, it may work, but you don’t get that- it’s like one size fit all, but it doesn’t” (NGT8).

#### Cost

Two of the 14 groups listed this among their top concerns, and three ranked this concern. It received 3% of all votes (9/288). Participants said that “There is lack of access to therapy due to the cost, with or without insurance” (NGT11) and “Insurance won’t pay for therapy all the time. My insurance limits me to 6 sessions or something” (NGT1),

### Difficulty with weight loss regimens

#### Difficulty in achieving weight loss

Four of the 14 groups listed this among their top concerns, and seven ranked this concern. It received 6% of all votes (17/288). Patients mentioned the following: “I did one time, when I lost weight, pain eased up. With COVID-19, I picked up weight and pain are worse now” (NGT3); “Had a pretty similar experience most of my life, able to lose 10 pounds, but not as much as I needed to get a major improvement in my arthritis. But then I would gain it again over the next few months. For me to switching to a plant-based diet has made all the difference” (NGT5); and “I have gained weight with the pandemic, the more the weight, the more problem” (NGT7).

#### Lack of motivation for weight loss

 One of the 14 groups listed this among their top concerns, and two ranked this concern. It received 1% of all votes (4/288). Participants from NGT5 said “I feel sometimes it is easy to get started losing weight. In the second week, it’s hard to move around and get tired and have to slow down. And getting depressed and getting something to eat, and weight loss, sometime, you want to give up” and “I start off good. But then I don’t lose as fast, as I should. Then, I get angry with myself. Then, I give up. I know it’s not good for my health. I guess it’s my mind playing tricks with me. Sometimes I don’t think I have the motivation” (NGT5).

## Discussion

To my knowledge, this is the among the first qualitative studies in a diverse patient population of knee OA focused on the barriers to the effectiveness of common non-surgical treatments (exercise, physical therapy, medication, weight loss) for knee OA. We explored patient experience, views, and opinions of why the current knee OA treatments are not working for them. This study oversampled African Americans and men with knee OA, since both these groups are under-represented populations in studies of knee OA. By prioritizing patient concerns, a hierarchical list of modifications/changes/interventions to improve patient adoption of knee OA treatments can be made. Several study findings merit further discussion.

Several medication-related concerns were rated as the top three concerns for barriers to treatment effectiveness in patient nominal groups. Among these, (B) limited medication efficacy (5 groups; 11% vote) and (C) medication not targeting the underlying disease (7 groups; 16% vote) garnered the most votes from the maximum number of groups. Additional medication concerns included (A) side effects (3 groups; 4% vote), (D) lack of the use of personalized medication or treatment (3 groups; 4% vote), (E) temporary benefit (3 groups; 6% vote), and (F) the fear of addiction/preference for natural treatment (2 groups; 3% vote). Not surprisingly, these medication-related concerns can lead to a feeling of medical uncertainty about knee OA and negative perceptions of drugs for its treatment, which can lead to lower medication adherence and a switch to complementary and alternative therapies [33], as noted in a recent German study. In another qualitative study, people with knee OA were reluctant to take medications to relieve pain and took them at a lower dose or frequency than prescribed [34]. Patient perceptions and attitudes to pain impact adherence to painkillers [34], which are among the most common medications used to treat knee OA.

Another major contributing factor to the limited medication effectiveness in knee OA is the belief that knee OA is an unmodifiable and inevitable age-associated disease [33], which was verbalized by several people in our nominal group study as well. Such beliefs can lower the confidence and trust in provider recommendations for any treatment strategy and in turn reduce its effectiveness and frequency of use.

This study also identified patient-perceived barriers to the effectiveness of exercise and physical therapy. Patients identified the (I) lack of motivation as the main barrier (7 groups; 12% vote) but also had concerns related to (G) the exacerbation of joint pain (1 group; 3% vote), (H) difficulty in doing exercises (2 groups; 2% vote), (J) technical challenges/lack of personalized exercise regimens (1 group; 1% vote), and (K) the cost of therapy (2 groups; 3% vote). In a recent systematic review of qualitative studies, pain and physical limitations; non-positive physical activity experiences, beliefs, and information; OA-related distress; a resigned attitude; lack of motivation, behavioral regulation, and professional support and negative social comparison with co-exercisers were the main barriers to exercise in knee OA [35]. Exercise behavior depends upon physical capacity to exercise, exercise beliefs, and other factors such as enjoyment, social support, priority setting, and context [36]. Identification of four exercise practices in knee OA (“long-term sedentary,” “long-term active,” “exercise retired,” and “exercise converted”) can be informative in identifying groups for an exercise intervention and modifying intervention to best benefit people in each group [36].

This study identified the following barriers to weight loss among the top 3 barriers: (L) difficulty in achieving weight loss (4 groups; 6% vote) and (M) motivation (2 groups; 1% vote). Our finding agrees with other studies of barriers that identified stress, depression, food craving, and situational barriers (eating out, travel, parties) and difficulty controlling appetite and diet, and perceiving that dietary control was not needed [37, 38]. The relative votes for difficulty achieving weight loss vs. motivation differ somewhat from that of a recent study that found 3-fold more people cited lack of motivation vs. knee pain as the primary barrier to weight loss for knee OA [39]. Due to weight loss-associated reduction of the risk of symptomatic knee OA and improvement in knee pain and function [40, 41], widespread obesity in US adults, and weight loss is highly recommended as a key management strategy for knee OA [18].

These study findings potentially advance the field. By enrolling minorities and men with knee OA, this study examined populations usually under-represented in knee OA research. The current study was comprehensive in that it examined all non-surgical knee OA treatments, i.e., medications, physical therapy, exercise, and weight loss. An in-depth discussion and prioritization of barriers by people with knee OA using the NGT helped to identify barriers of higher relevance/importance. These can now be potential targets for behavioral interventions and/or quality improvement (QI) efforts.

Findings from previous studies and this study can help with the development of effective and patient-acceptable knee OA interventions. Key reasons for the failure of knee OA treatments were the inability to exercise or lose weight, a lack of motivation for physical therapy, exercise and weight loss programs, a limited efficacy of medications in knee OA, and the current medications not targeting the underlying disease. Future multifaceted interventions for knee OA should also address the following aspects: (1) ways to achieve effective patient-provider communication, regular assessments, and flexibility for better and appropriate treatment planning [42]; (2) methods to target providers and family support, time, resources, and clinical characteristics that influence the treatment choice [43]; and (3) lifestyle behavior change for encouraging knee OA exercise that incorporates patient-provider shared decision-making, motivational interviewing, positive factors for building confidence, planning, and goal-setting [44].

Key study strengths were the inclusion of a community clinic-based sample, African Americans and men with OA. These study findings must be interpreted considering its limitations. Due to the under-representation of African Americans and women in previous qualitative studies of knee OA, these groups were oversampled in this study.

Study findings must be interpreted considering the study limitations. A potential limitation was that men with knee OA were few, which mirrors its epidemiology in the USA. The mean knee OA disease duration was 8 years, and most demographic and clinical characteristics are like other knee OA cohorts that make these findings somewhat generalizable. Our study findings are not generalizable to the population of patients with OA that have their first knee OA-related consultation with their general practitioner or internist and need to decide on what modality to try first, since all our participants had tried one or more modalities. Future research should examine these populations.

## Conclusions

In conclusion, this qualitative research highlights the key patient beliefs and experiences related to barriers to knee OA treatment effectiveness. Patient experiences and attitudes should be considered when implementing disease management options/pathways for people with knee OA, to make them more effective. This theme was noted in a recent systematic review of qualitative studies of patients with knee OA [21]. It is important for public education campaigns to address how the current knee OA treatments can help to manage knee OA pain and associated functional limitation, which predominates the lived experience with knee OA [21]. The current study reinforces these findings but also adds new knowledge regarding the relative prioritization/importance of these barriers to treatment effectiveness.

## Supplementary Information

**Additional file 1.** All themes and related main discussions for reasons for the failure of the current non-surgical treatments for osteoarthritis from all nominal groups with the number of votes within each nominal group (Why do you think current treatments (medicines, physical therapy, weight loss, exercise) for osteoarthritis (arthritis due to loss of cartilage; wear and tear arthritis) of the knee joint do not work?).

## Data Availability

We are ready to share the data with colleagues, after obtaining appropriate permissions from the University of Alabama at Birmingham (UAB) Ethics Committee, related to the HIPAA and Privacy policies.

## Abbreviations

NGT: Nominal group technique
UAB: University of Alabama at Birmingham
OA: Osteoarthritis
ACR: American college of rheumatology
AF: Arthritis foundation
NSAID: Non-steroidal anti-inflammatory drug
ICD-10-CM: International classification of diseases, tenth revision, common modification
