# Supplementary material for: “I wish it had a place to go”: a nominal group study of barriers to the effectiveness of non-surgical treatments for knee osteoarthritis inclusive of minority populations
Source: Arthritis Res Ther. 2021 Dec 1;23:291. doi: 10.1186/s13075-021-02676-8 (PMC8633910; doi:10.1186/s13075-021-02676-8)
Supplement: Supplementary file 1 — Additional file 1. All themes and related main discussions for reasons for the failure of the current non-surgical treatments for osteoarthritis from all nominal groups with the number of votes within each nominal group (Why do you think current treatments (medicines, physical therapy, weight loss, exercise) for osteoarthritis (arthritis due to loss of cartilage; wear and tear arthritis) of the knee joint do not work?). [file 13075_2021_2676_MOESM1_ESM.docx]

**Appendix 1.** All themes and related main discussions for reasons for the failure of the current non-surgical treatments for osteoarthritis from all nominal groups with the number of votes within each nominal group (**Why do you think current treatments (medicines, physical therapy, weight loss, exercise) for osteoarthritis (arthritis due to loss of cartilage; wear and tear arthritis) of the knee joint do not work?**)

| **Theme** | **Votes** |
| --- | --- |
| **NGT 1: 2 people: 2 F; 1 White, 1 African American (12 votes)** | |
| A. The current treatments I had, I had shots in my knees, gel treatment/shots, cortisone shots, repeatedly.   - I was thinking I had shots in my knees, knees were still bad, it helped one time, but after so long, you have to get some more shots - The cortisone shots do help - The gel injections did not help at all, so I had to go back to the cortisone shots - It’s a temporary thing with the cortisone shot. I want something that would help for a while, not just 2-3 hours. Something that would help me to get around better. - You want them to last longer. - When I first started taking the cortisone shots, they lasted like months, almost a year. Then the pain came back. Then I got another cortisone shot, over the course of time, they lasted less time. Usually, they drew the fluid out and then give me a shot. The last time, they couldn’t draw the fluid, “fluid crystallized”, and it only lasted 3-4 months. - The last time for me, it lasted 6-7 months. - I would like it to be longer than 6-7 months, may be 1-1.5 years; it just should last longer than 6-months. - Of all the treatments, I like the injection the most. It would be nice for it to last longer. - The pain of the injection is not as bad the pain in my knee; it just stops working for me, my knee would swell up really bad. - Nothing worked as good as the cortisone shots. | 0 |
| B. The weight loss would help, but it’s not easy   - I have been trying to lose some weight, and they said it would help, and do a lot of exercise, but the exercises are painful, when it’s bad. - I was looking at the option that if I lose the weight and it would help. And that’s what I have been trying to do. But with the pandemic, you can’t exercise, and a lot of things were shut down - I believe it can help some. The doctor told me that it hurts because you lose your cartilage, bone on bone, keep the bones from rubbing from each other, by losing weight. I haven’t lost that much weight. It’s hard to lose weight. I am 60 lbs. I believe losing weight should help. - Weight loss cuts down the pressure, but the bone on bone | 6 |
| C. I was in therapy for a long time, have to do it for 6 weeks- have to do it continuously, it’s tough work   - It did help, but I could not continuously do the therapy, pain would come back. - Therapy was good. - It probably will work, if you were able to do some of the exercises that they have. - I don’t think it’s a permanent cure, but it helps, you are moving, you are mobile. It helps the pain. But you have too continuously do it. - It’s painful to exercise. - It’s hard to put down step, hard to walk during therapy | 0 |
| D. My insurance doesn’t allow me to do therapy as long as I would like to do it; Pandemic makes the physical therapy difficult to do   - It’s like the pandemic, when you go somewhere you have to get in a line, you have to stand on your knee in a long line, and it would really hurt. You have to wait to get in. It puts you back where you were, before it happened. - Pandemic caused a lot of problems; you can’t have anything done. | 3 |
| E. Insurance won’t pay for therapy all the time   - My insurance limits me to 6 sessions or something. | 0 |
| F. I believe that most of the medicines don’t work, because it’s bone on bone, there is no cartilage, there is nothing to stop it. I can hear it when I walk. So far, the medicine is concerned.   - It’s temporary; medicine is good, but it’s temporary, you have to take it all the time. - If I am sitting it doesn’t hurt, it only hurts when I am walking or going up and down the stairs. - About the medicine, some medicines put you to sleep, and when you wake up you are in the same pain. - The difference between pills for the blood pressure, it helps keep your BP down. With pain, the pain is coming back when. It could be a cream. - I don’t like taking any kind of pills. - The only thing I was prescribed for pain was ibuprofen. I have a chronic pain condition anyway, I take a pill for that (fibromyalgia), that helps | 0 |
| G. Pills have Side effects   - I want to be alert during the day, if the medicine makes me groggy, I don’t want it (cyclobenzaprine; may be another medicine for my osteoarthritis). - Ibuprofen makes me groggy- side effect | 3 |
| **NGT 2: 4 people; 4F; 1 White, 3 African American (24 votes)** | |
| **A.** Problem doing Exercise:   - I do exercises at home. I can do hand and foot bike for 30 minutes. - I can’t do bending over due to pain. - Can’t do due to other conditions. | 0 |
| **B.** Exercise makes knee pain worse:   - I feel like certain exercises may actually exacerbate the pain. So, I am not real sure, exactly what I should do. I swim every day for 30-45 minutes, and this includes bending my knees, which I believe increases the pain. But I am moving my entire body and feel this must help keep me strong. My knees hurt when I get back from swimming. I don’t want to do something that hurts more, I am wondering whom to ask. I can google it on the computer, or ask my primary care MD, but she doesn’t do therapy, I could ask one of the therapy people at HealthSouth, to ask them what they think I am doing. I don’t want to do something that makes it worse. - If I don’t swim, I walk treadmill for 15 minutes | 2 |
| **C.** I got an injection that relieved my pain, and it lasted only 3 months   - Work for a while, then it won’t work - Mine didn’t work from the beginning. I didn’t get a chance to get used to it. - I took a series of injections that didn’t work for me (? Hyalgan/Synvisc) - When my doc gave me cortisone shot in both my knees, they worked great. But then all of a sudden, they didn’t work anymore, that’s when I decided to get a knee replacement - I heard about stem cell therapy, not sure it works or not, but it was not covered by my insurance. I don’t want another replacement of my knee, that’s one of the most difficult operations I ever had. | 4 |
| **D.** Medicine helps some, but pain persists:   - Because of changing my diet, my tolerance for medicine- it’s ineffective for me - Medicine helps some, but not much, I am constantly in pain - As far as medicines are concerned, I take meloxicam and gabapentin, this does not seem to eliminate the pain that I have. Creams do not seem to help when you put them on your knees. I have been very disappointed that can’t find something that really helps with the pain. - I feel like that I am taking more medicine than I should be taking. Especially the medicine for OA knees, does not seem to be helping at all - I tried Voltaren, it was very expensive. I don’t think the creams really help - Trying to keep the pain down, but they don’t seem to help. So, what else to do to alleviate the pain - Recently my doctor told me to try CBD oil, when I tried the lower strength, it didn’t help. I need to try the higher strength, but don’t know how well it will work - Treatments I had didn’t work. | 7 |
| **E.** Medicine has side effects:   - it did better when I could take anti-inflammatory medicines (naproxen). Can’t take it due to the heart failure. - I started taking hydrocodone and continued it until they started lowering my Tylenol. Then started taking oxycodone. - The majority of the medicines, anti-inflammatory medicines, I can’t take it at all, due to my stomach. Not over the counter not the prescribed ones. - The only thing I can take is narcotics for pain. You can’t take narcotic continuously and be able to function normally. | 4 |
| **F.** Weight loss is difficult:   - it fluctuates, because sometimes I have to take steroids for flare-ups of my RA. Inability to be able to exercise - Weight puts a stress on the knee and puts a stress. I try to lose weight - I was trying elliptical machine. - I did one time, when I lost weight, pain eased up. With COVID-19, I picked up weight and pain are worse now - Lost 10 lbs. during the therapy at Spain rehab, my balance got better, and knees weren’t giving away; started having trouble with breathing and stopped going - I only weigh 100 lbs., it’s not extremely important for me to lose my weight. If someone is heavy, they must lose the weight to put less stress on the knee. | 3 |
| **G.** Therapy: because of the pain, I am unable to do it, as I should   - That makes everything ineffective - Whenever I did therapy, it was very painful. If they gave me something for pain, pain overweighed the medicine at the time of doing therapy. My therapy increases the pain. They would have to take me out on a wheelchair. - Lost 10 lbs. during the therapy at Spain rehab, my balance got better, and knees weren’t giving away; started having trouble with breathing and stopped going | 2 |
| **H.** Therapy making OA symptoms worse:   - It was extremely painful. I think it’s necessary. I was at HealthSouth and felt they had too many patients at the same time. After I finished the therapy, I got sciatica, which was on top of getting through the therapy. - I had therapy at Spain rehab helped me, helped my balance. | 2 |
|  |  |
| **NGT 3: 3 people; 1 M, 2 F; 1 White, 2 African American (18 votes)** | |
| **A.** I feel like it’s not working, because I am not taking the right medicine. The pain is still there. I feel like my medicine should be changed.   - It’s consistent with me too, it just takes the edge off - I feel like the medicine don’t work, I work at a School, and I am constantly walking. The pain in the hip and all the leg. I take the medicine, and I still have the same problem. I feel like that I am not taking the medicine. I have the pain at home, pain at work - Sometimes I work, I had a lot of pain. I had to change my shoes, to get better support. - I also take injections | 2 |
| **B.** The missing cartilage is still missing. Activity and weather still affect the pain. Until something is done for the cartilage, the pain will continue.   - I agree - I have some cartilage wear, and I am having surgery done, it was recommended for me - Seems like we still have to function. The level we have to function is diminished severely by what I call “sore day”. The solution is to take more medicine. No cure to stop the cycle. Until we do that, it’s a continuous battle that we can’t win - My knee is bone-on-bone, that’s why doesn’t work all the time - I feel like I have a lot of walking that’s probably why it does not work. | 3 |
| **C.** Pain remedies only address pain; Treatments are only prophylactic, and only for the most difficult days.   - It’s true. - Is there a treatment for the arthritis itself that suppresses the arthritis to the point? Is there anything on the table, or in the future, that we can do that - Part of the treatment that I do have to do with putting ice packs, or elevate my knees, on top of taking pain remedies and anti-inflammatories. It just seems there should be a better way to do all that. Why can’t we find a way to stop the arthritis? - Whenever I am in a lot of pain, I try heat and it helps - Once I sit down when I am in pain, the pain goes away. When I get up and start working again, the pain comes back. They say use ice packs, heating pad. Ice packs don’t help; heating pads doesn’t do much. Pain is back when I am walking or at nighttime. - Until we address the cause of the pain, treatments are not the cure - Another aspect is that if you are applying ice packs, and heating pads and elevating legs, you are not doing your job- that interference with life is a big issue | 5 |
| **D.** In order to function, I have pain and swelling, so there is some trade-off.   - The more you medicate the pain, the less capable you are of high-level function. Less able to concentrate, not only the pain, but pain medication interferes with it. This is just as big a problem as to have to deal with the pain. - We have this opioid crisis going on where people are taking it for pain. We still don’t have a pain solution, that doesn’t interfere with your life. - I would like to see treatment of the source of pain. | 0 |
| **E.** Sometimes we have more weight that causes us to have more pain.   - One time I was on prednisone, and it made gain me a lot of weight. Once I would lose weight and was exercising, I know the pain was less. - The one thought I have is that if they lift something heavy, as part of your job. Isn’t that pretty much the same thing as having gained more weight. - Moving heavy objects or heavy groceries. - We don’t eat the right type of food, we drink a lot of sodas and don’t eat work | 4 |
| **F.** We don’t exercise the way we should exercise   - Sometimes exercise causes more pain, and that’s why don’t exercise. Is there a way to overcome that cycle? If you lost enough weight would you overcome that cycle? - Sitting in a chair and doing the stretches, we can do that. Easier than a bike or running. - If you are moving too quickly or too much and the cartilage is missing, you are really causing inflammation when you are exercising, at least on the temporary basis. May be the exercise should be more directed to ROM, rather than enough exercise to lose weight - Mine is not for losing weight but to keep good muscle tone. - Sometimes jogging and treadmills without wearing knee support causes pain. | 3 |
| **G.** The weather- cold weather   - Nothing works as well when it’s cold | 1 |
|  |  |
| **NGT4: 8 people; 1 M, 7F; 4 White, 4 African American (48 votes)** | |
| **A.** After your cartilage is gone, it’s just bone on bone, I don’t think any treatment other than the surgery will work   - I think that’s a true statement, because nothing can replace it. - You can’t replace what’s already gone - I went through the gel injections and they didn’t help at all, because it was bone on bone - I went to physical therapy and scope, and none of that helped at all, until I got the knee replacement. So far it is doing well - It seems to be reasonable direction. - It’s too bad we can’t catch it before it gets too bad. - A lot of it is because you don’t want to go through that surgery. - Because of the loss of cartilage, you have bone-on-bone and that causes the pain. Medicines do not work because they don’t duplicate the structure of the joint. With being bone-on-bone nothing to absorb the shock, so can’t recreate it. With the loss of cartilage, you have problem with lateral movement. - I think doctors want to medicate you more, than listening to you tell them about your problem. With bone on bone and cartilage, you need replacement - Once you lose stuff in your body, you can’t replace it; The original is what God has given me, mankind has some options, but it’s not the original - Science has not come up with a way to manufacture cartilage, other than a total replacement. - I think that this is just normal wither in the knee joint. As you age, this joint gets naturally withered down. | 21 |
| **B.** According to me, it’s just trial and error, some meds may work for some people and some don’t; they don’t last a lifetime; I have taken medicine and I have had to go up my medicine, because half the time it doesn’t work and when it does work it does not last long.   - Not all people are the same; and not all treatments work the same way with the people - I think it’s the mindset, if you don’t think it will work, it won’t work - The main thing is that you are worried about liver and kidney, about taking so much pain medications. - What might work for one may not work for the other person - They don’t last long enough - I feel a lot of people are afraid of the medication, because you might get hooked on it. People are afraid of that, and what it might do to them. - Sometimes your body get used to that medicine and you have to increase the dose to make it work the same. - I don’t think the medicine work. | 8 |
| **C.** Medicine side effects to certain meds.   - Sometimes on the medication it causes me so much side effects, make you groggy, and sick - When I take some medication, it gives me a rash, and its more problems that it is worth - Some cause nausea; some cause bone loss - Some make you so tired you can’t work. | 4 |
| **D.** Being too overweight, the exercise can exacerbate the wear.   - Does it not make the wear faster, if you exercise? | 3 |
| **E.** I have had several injections that don’t work   - In my case, when I had the injections, I got up on my knees too fast, I didn’t give it enough time, I think - I think it varies from person to person. - I started injections when I was 23 years, I get 2-3 days of relief, when I get injections. It’s a different type of medicine each time. - Sometimes I waited too long between the injections. By then it’s so severely inflamed. It doesn’t work as well. - Doc says you can come every 4-months. Sometimes, it lasted only a couple of months. - Why did they wait so long to give you injections in-between? I think it’s an FDA thing and insurance won’t cover. They have to get approval to do these injections. - The injections I received in the knee did not help; my right knee was bowing in, it prevented me from my daily activity, there was no medicine that would help it other than total knee replacement. | 0 |
| **F.** I am not able to exercise.   - My legs aren’t strong enough for me to exercise. I was a cheer leader, and did gymnastics, and now I don’t have the strength to exercise. I couldn’t even swim from one end to the other end because my legs were so weak. - Some people hurt so much, that they don’t want to exercise, because it causes so much pain and that’s one reason why they can’t lose weight. - After I exercise, my knees hurt more. - Physical therapy: I have done it before, and I don’t see it help/benefit, it causes more pain by doing therapy. - MY knees hurt when I walk a lot, makes exercise difficult to do. - When I exercise and I don’t have the right shoes, my knees will hurt - You can’t exercise and do therapy because it causes a lot of pain. - You can’t get to exercise, and you are trying to lose a lot. | 3 |
| **G.** I have taken medicine and I have had to go up my medicine, because half the time it doesn’t work and when it does work it does not last long.   - My problem is that I don’t take medicine on a consistent basis. I wait too long, and the pain gets too far out of hand. - I take my medicine on a timer. When I don’t take it consistent, I hurt more. - I take my med in the AM and it wears off before I have to take it again. Pain is worse. Not Controlled. | 4 |
| **H.** If the medication doesn’t work within the first couple of days, a lot of people will quit taking it, because they don’t see the benefit.   - My doctor told me that it will take a month to get relief from medicine and I took it for 2 months and didn’t get any relief. - In my case, I am bad about taking medicines. - When I start a new regimen, I would give it 2-3 weeks and then talk to my doctor and let him know. - When my pain medicine stops working, I go with the Voltaren cream. | 3 |
| **I.** You are not on the proper regimen to get relief of pain.   - Following the doctors’ orders- you are not following the orders - If you skip a dose here and a dose there, it will take a while for your body to get adjusted. - Expect relief too fast. | 2 |
| **E.** *Some medicines make the wear of the joint quicker*   - To me it’s more of a reaction to the medication. Steroids is that what I am thinking of it. - Prednisone can make joints wear quicker - Your body can only take so much, if overweight, your body can’t function properly - I have lost 65 pounds in the last few years, did not help my pain at all. I lost muscle mass. - I feel I am overweight. It seems like even though I am overweight, I still hurt. I have hard time losing weight, so I just gave up on it. - When I start losing some weight, I felt better. - I lost from 210 to 172 pounds and it didn’t help my pain at all. - When you lose a lot of weight and it is not helping. When the joint has gotten to the point to the joint being worn out completely. So, it’s not going to help. | 0 |
|  |  |
| **NGT 5: 4 people; 2 M, 2 F; 2 White, 2 African American (24 votes)** | |
| **A.** Medicine: The reason I think it doesn’t work very well, because I take other medications. And I have to take this medication to counteract the other medication, so that It won’t affect my liver or my kidney. That’s one of my opinion is why I think the mediation doesn’t work.   - I agree with that. I take many medicines. I have lupus, which doesn’t help. Have to have blood work and eye exam, it causes other problem. - With medication, I have high BP and cholesterol. They ask me to take pain medication, but it can affect my potassium, I had to go to the ER. - I take insulin 7 units to help joint pain, once a week. Used for gout. | 7 |
| **B.** Weight Loss: MOTIVATION: I feel sometimes it is easy to get started losing weight. In the second week, it’s hard to move around and get tired and have to slow down. And getting depressed and getting something to eat, and weight loss, sometime, you want to give up.   - In my experience, I go on a diet routine, then give up and stop. Then I start normal eating, and I stop exercising for some reason, and then I have more weight gain. - I start off good. But then I don’t lose as fast, as I should. Then I get angry with myself. Then I give up. I know it’s not good for my health. I guess it’s my mind playing tricks with me. Sometimes I don’t think I have the motivation. - As far as weight loss, weight loss works for me. If my weight goes up, I know I have to lose weight. And that works for me: I believe weight | 4 |
| **C.** Weight Loss: difficulty getting adequate weight loss:   - Had a pretty similar experience most of my life, able to lose 10 lbs., but not as much as I needed to get a major improvement in my arthritis. But then I would gain it again over the next few months. For me to switching to a plant-based diet has made all the difference. | 2 |
| **D.** Physical Therapy: TOO MUCH, cause too much pain: which I experienced.   - I went to the therapy; it all depends on who you have. Some therapists don’t push you enough and some push you too hard. “You can do few more and sometimes it does more damage” - I experienced that. It’s not who you are. It’s who you see. I went there right after my knee scraping. “We feel that you can do more, and more”. I can barely walk and get to my truck when I leave somedays, I am feeling worse than when I walked it. - Certain days they are doing much less. I could have done it with a ball at home. - When I tell you I can’t do it, let me tell you when I can do it. You don’t know my body. - Some therapists push you more. I am limping more when I come out of therapy. I can do my exercises at home; I can do better than the therapy. I thought therapy is not an option for me at my end. - I had 2 types of therapists. One trying to increase my strength, that helped me a little, I had more pain related to those exercises. When I had pain, I didn’t keep it up. Another therapist’s aim was to help me stretch more and help me walk more optimally. That was very effective. It was little painful, but not serious. It just worked better for me. | 2 |
| **E.** Physical Therapy: Variable, varies by who you go to in PT   - Because they are able to do more, they expect us to do more. They want us to go well, so they will do a good report. So that company will look good. - Different opinion of each therapists - I feel like some care, some don’t. Some there to just make the hours and some really there to help someone - I have never had a therapist tell me that they were doing what they were doing was based on scientific evidence – may be was anecdotal. | 1 |
| **F.** Exercises: Not knowing: While I do feel like I am ok when joints loosen up, I feel like I am 17 and then go out there and overdo it.   - Some days you wake up and you are feeling good. “I can do a 1.5 mile instead of a mile, the extra half mile, you do more damage”. You can overdo than what your body is telling you. We hurt ourselves doing it. - It would help us a lot if there were ways while we are exercising, we know that we exceeded our limit. You can’t until after that you had done too much. Help us match exercise to what was really therapeutic for us. - I had a personal trainer for several years, showed me things to do to help me my arthritis. “If it hurts don’t do it, it will do more damage” - Also heard some trainers say, “no pain, no gain”, but that’s not right. | 0 |
| **G.** Exercises: lack of equipment:   - Pretty much I have experienced all of it. The only thing I have problem with is that you don’t apply the thing when you go home. I am not able to do the same thing I do when I get home. - We don’t have the machines that they use at the therapy. - Trouble remembering all the exercises, I need to do. Sometimes even complicated to have written exercises. | 2 |
| **H**. EXERCISES worsen pain: Again, the exercise. I have done exercises, but if I overextend, it makes it worse. | 0 |
| **I.** EXERCISES: temporary relief:   - For me exercise has helped a lot but want limited its effectiveness is that you can’t exercise all day long, and it helps while you are doing it. | 0 |
| **J.** Injections: Incorrect Spot: First of all, the injections, I have had many. Most of them don’t work. Sometimes I think injections are not in the right place.   - December worked well, in June pain got bad, they did the injection. Sometimes injection would work, sometimes not. Was wondering, whether it has to hit certain spot. Wondering why it works sometimes and not the other times. - Pulled out the fluid, 35 ml. then he injected in my joint. Two weeks later, still hurting, and hopping down the hallways. - One experience with the steroid shot. I did a shot for the hip and then for the knee joint pain. First 2 steroid shots worked and the third one didn’t work at all. I can’t explain why. Maybe body chemistry. - Always worked | 2 |
| **K.** Overall, the problem is hereditary. And nothing would work.   - Therapies are mostly trying to alleviate symptoms, very few are designed to slow down the loss of cartilage - Since it’s hereditary, nothing works - No cure - Medicine, they help, but are limited by side effects, like upset stomach and kidney toxicity. But they can’t create new cartilage. - Medications are only a band-aid and doesn’t repair the damage. - Therapy: I have had both PT and injections. Both helped but they cannot create new cartilage | 1 |
| **L.** By the time you start you start any treatment, it’s too late | 3 |
|  |  |
| **NGT 6: 4 people; 2M; 2 White, 2 African American (24 votes)** | |
| **A**. I believe that the treatments work on a limited basis to treat the symptoms   - I don’t know. The treatments are the treatments. They don’t make it go away, they make it better - And it doesn’t last long-enough. - If I go get my treatment, do exercises, or my insoles, it relives a little while but not long enough. - Physical therapy works you strengthen the muscles around the joint/problem areas, it’s better, it doesn’t make it go away. - When I was working, I had PT, it didn’t do much. As long as I was doing the PT and was at home. As soon as I went back to work, had to stand on concrete floor all day long, PT wasn’t good once I went back to the work. Up to the person, and the job they have. | 8 |
| **B**. Motivation/Time: When you get back to the real life, you can’t put so much work into, then when you are off.   - It’s easier when you have an appointment time, than you are working on your time, you put yourself last. - Working with therapist they are holding you accountable to do the work. - I have 2 parents and a child to take care. - You must be disciplined to follow the treatment to get the benefits | 0 |
| **C**. Motivation/Pain: It’s hard to get motivated, when you are in pain.   - Some of the PT is painful itself, if you are in pain, you don’t want to add more pain - Right now, I like to go bike riding, but my knees are not stopping from bike riding, my foot is stopping that - Pain can stop you from doing things that you love to do. - I can agree with that as well. It does stop you riding the bike. My knee bothers me 80% of the time. It hurts me to have my knee bend. One of my bikes is a heavy bike, almost the knee wants to give up, I don’t ride like I normally I do it normally. - This morning I was going to do Yoga, because my knees were hurting, I had to postpone it. That’s a lot of my problem. - The world would rather mask or try to do a quick fix, rather than truly fix the problem. Once the cartilage is lost, there is really not much they can do, they can make the muscles better. - Like for me, when my knee is worked on, it makes the pain move to my foot or hip. | 8 |
| **D**. However, once you lose the cartilage or it is damaged, then these treatments do not do anything to regenerate the cartilage.   - There is a root problem, damaged or lost cartilage. Therapies and medicines don’t fix the problem - In other words, treatment help to deal with symptoms, but they do not fix the root problem. - The bone structure is hereditary, in some cases, for instance my great-grandmother and great grandmother and all her sisters are bow-legged, and it passed on to me, and it’s passed on to me, and that’s my problem, my doctor told me. - I guess, just hereditary, nothing I can do other than surgery. - Almost bone to bone, cartilage is gone. - Also, may be looking at something at genetics, both my parents are suffered from arthritis, and my grandparents too, I was meant to have arthritis. | 5 |
| **E**. Like for me, it’s hard to lose weight to be able to take off some pressure off my joints   - I am on the heavier side right now, and it is due to my age and insulin resistance. It’s hard to get weight loss. I almost worked up to almost 4 miles, but I hadn’t lost any weight. | 0 |
| **F**. Medical profession should look at other alternatives, rather than man-made medicines, like natural medicines.   - There are not very many clinical research studies on natural remedies, or homeopathic, when you ask for studies like that, people look at you like you are weird or something. People form the healthcare team - It’s kind of like, well this pill or cream didn’t work, let’s try you on something else. - You have to pay out of pocket to try anything natural. Difficult when you have nowhere to start or don’t have any resources. - You either have this or that- it’s basically the same they are all trying the same thing that medical professional has already tried on you. - I do use natural meds between times when my prescribed meds are not working. When I take different types of herbal or natural meds, it seems to work better. - Natural meds I tried is the CBD oil and paraffin wax? I use it moderation. I have used it for so long, I get right back to the pain. | 0 |
| **G**. I also have lupus and they say that’s part of my problem too- trouble with taking knee arthritis medications due to lupus.   - They said lupus comes with arthritis too - I am used to pain so I just deal with it. - I have been to therapy; I do exercises sometimes. MY knees still crack and pop. Even moving on the recliner, it pops and hurts. - Because of my lupus, I am allergic/side effects to a lot of medicine. If I take it to relieve pain, it causes skin irritation, or other problems, like my other medicines that are working. | 1 |
| **H**. My arthritis gets worse with daily activities, because I am active, and that’s why meds don’t work well for me.   - Some of the meds for work for me; some do not; they work for a limited period of time. I do heat and ice packs on daily basis. My body stays in pain. You can hear my bones crackle in the next room when I move | 1 |
| **I**. My doctor has progressively gotten more aggressive with my meds based on my daily activities and my pain level; I don’t think that’s getting it.   - To me that says the medicines work, but when you are activity level increases to a point, that they don’t work anymore - When I can take medicines for a while, and it works, and then it quite working all together. | 1 |
|  |  |
| **NGT 7: 4 people; 4 F; 2 White, 2 African American (24 votes)** | |
| **A.** May be because I am not on the right diet- tomato, pasta   - Do less sugar, drink more water, eat more vegetables, do beef instead of pork. Do less alcohol - If I do less of this, less inflammation, treatments work better - I do agree on the sugar, if I eat too sugary, I feel it on my ankle, that’s got to be too sweet, because I am feeling the effect of it. - If I have any salt, my ankles get big - My knee is swollen all the time, you can feel fluid. I don’t know what I should do to help. | 3 |
| **B.** Prepare your own food instead of eating out all the time.   - Because you prepare your own food you know what’s going in your body; if you do too much salt, it holds inflammation in your body, makes the treatment less effective - It is possible to eat healthy, if you do have to eat out. | 1 |
| **C.** Routine: Exercise at least 15-20 minutes a day   - Loosens it up, loosens the nerves; once we get hold of these nerves in the knees, once we loosen it, we can be free to do things - Once I start it, I may start slow, once I start, I can get going. You will be good about this | 5 |
| **D.** Weight loss may not work sometimes: Watch your weight   - I have gained weight with the pandemic, the more the weight, the more problem - Weight loss works by decreasing the weight on your knees - I lost while I was in the hospital, almost 40 lbs., but I couldn’t tell if it was better; maybe it would have been worse without it | 3 |
| **E.** Personalized treatment with medicines: Because medicines haven’t been tested enough.   - When the person goes and they get the diagnosis, they will just come out and give you a medicine. They haven’t been tested long enough that it will work well for me. - There are so many different medications that are available, what might work for one may not work for another person; it could come back to side effects. - They tend to give you more medicine, you get side effects. 2 months ago, I was on a medicine, was on it for 7 days, I never heard of it. There was something about the name I didn’t like, then I had to stop that since I broke up in a rash with it. | 3 |
| **F.** Personalized Therapy, weight loss and exercise plan:   - They will help, but medical doctor doesn’t give you a remedy for it. - They tend to give you more medicine, you get side effects. 2 months ago, I was on a medicine, was on it for 7 days, I never heard of it. There was something about the name I didn’t like, then I had to stop that since I broke up with it. - It’s all about the individual!! - Sometimes I have a doctor that will hand me a diet plan to lower cholesterol that might also help the knee pain, lower in gluten. “But wait I am allergic to half of that, it doesn’t reflect my personal need” | 1 |
| **G.** It doesn’t build your cartilage   - Can anything re-build the cartilage truthfully? If they can’t build cartilage, they won’t work as well. - I Know medicine would build it- I think medicine tears it down even more. | 3 |
| **H.** Lack of motivation/It takes commitment   - You have to commit to PT; to exercises; to special diet; to our treatment plan- if you don’t commit it will not work 🡪 limit o it your insurance company doesn’t allow or cover - You really help in getting committed to treatment. There are so many things that can distract you from your daily routine - I need motivation sometimes. - Because I don’t exercise enough | 5 |
| **I.** Physical therapy can make you hurt   - I understand the importance of it, but at the same time, it can really hurt, when you are in pain - Because they are trying to build the muscle and there is muscle you have never used. - I have PT a lot on my knee, it builds muscle around the knee to make it stronger. - How hard they go into it? Some therapists are really aggressive | 1 |
| **NGT 8: 3 people; 3 F; 3 African American (18 votes)** | |
| A. Pain interferes with the ability to do therapy - no immediate relief with PT or meds   - When you do PT, you are already in pain you have to get past that pain to get any results - Also depends on the individual, something that works for one person does not work - It’s always there - It’s always there - It would be to the point that it hurts and makes you cry; it wakes you up -> despite PT and meds - It depends on the diagnosis- you can have; your muscles and cartilage are deteriorating on you, you will have the pain - It doesn’t work - Because you have no protection from the pain – so the pain will be there | 2 |
| B. It’s the general medication or regimen for everybody; like you are doing one pill for everybody   - That’s true for me too; they treat everybody the same. I have been going through this since 2016, feel worse, you can not relax or feel better. I use cases of bio-freeze 🡪 then got the injections last year, could walk 2-3 months, but the steroid shot makes you gain weight. - Shots may work for her; it didn’t work for me 🡪 it did not work for me. There is no treatment that is guaranteed to work | 3 |
| C. Individual PT and exercise:   - It should also apply to therapy and exercises as well. Some people with medications, it might not work. - It’s an individual thing. I have tried, you can’t do a bunch, but can’t exercise when you are in pain. - It does not relieve anything, when you exercise and then go to the bed, you are hurting worse from exercise and the arthritis - Everyone’s body make-up is not the same, so this will help - Just in general, the exercises they want you to do, it’s the same plan they are giving to everyone- may shorten the time, then it’s the same treatment - If you tell them you can’t do it, they think you are faking - If you had an individual program prescribed for your bodyweight and body size, it may work, but you don’t get that- it’s like one size fit all, but it doesn’t | 3 |
| D. With exercising with medication, might give them a sense of relief.   - With me usually if my knees are bothering me, I would do exercise, medication and soak bath. Heat soothes me. I feel better - It just doesn’t work it just didn’t work | 0 |
| E. The damage is already done; There is nothing to help you healing of your knee, you have no cartilage   - You are beating a dead horse. If the damage is already done, how can you relieve the pain - If you have tried everything like therapy, what’s the next step. - Because you lose it or you have lost the cartilage that is in our knees, you are technically rubbing bone to bone, from what I have been told - You are following the regimen; you are still not getting the relief. It’s just not going anywhere - It’s sort of like you are trying to do a quick fix, and not a permanent fix | 2 |
| F. Side effects with medication   - One is weight gain; medication makes you gain weight; prednisone being prescribed for RA - Some side effects are totally different than others; some do not have side effects, and some do - With me, I am blessed, I have not had any side effects from any of my medications, but I have seen other people who had side effects, like diarrhea, skin rashes, headache and others. - If you have more than one diagnosis, you will have some kind of side effects. There are different side effects - You don’t which one of causing the side effects, you are taking so many. - I was on lisinopril had started my RA injection, somewhere I had a side effect –sitting at home, couldn’t talk or breathe, lips and swelling had started swelling, went to the ER, my lisinopril was there for 5-6 years; was on MTX, Humira- stayed on ICU for 3 days | 1 |
| G. I feel like pain comes back after the treatment in 2-3 months; you have to do something to sustain the treatment.   - That’s the first time I had the injection, that’s how long it lasted. I could walk without pain, I could sleep. Then the pain came back, pain was worse. I was getting injections every 3 months. - Injections only last 2-3 months; pain of the injection, and maybe it’s the mental thing; unpredictability of the relief duration that makes this a problem - I take an injection for migraine, 1 shot every 30 days; no such format with injections 🡪 treatment plan is lacking about the frequency of the injections - I would think it should be as needed; I can tolerate pain, depends on the individual, when they should take the steroid injection again. My knee injection was last year, haven’t had one since. | 1 |
| H. I think there is a lot of natural remedies that can help rebuild cartilage without all of the medications that told us to take   - It depends what you are taking in the natural products, that can lubricate/rebuild the muscles, cartilage: Boswella, it really helps me “crackle” less - These can Improve effectiveness of other treatments - I never took any, so I don’t know whether it helps or not. - Doctors don’t give you that information: Some may know, but they have to push the prescriptions – alternative medicine training in that field should be part of the medical training - You can always have a booklet in your office about natural remedies | 6 |
|  |  |
| **NGT9: 4 people; 1M, 3F; 1 White, 3 African American (24 votes)** | |
| A. The reason why medicine doesn’t work, you have to keep constantly taking and there is a price point   - Some people may not have insurance, pay out of pocket, have a high co-pay, deductible - You can always get a generic brand, but it may not work as well as the main brand - The civilian doctors offer a discount card to take to your drug store; ask your doctor for samples | 3 |
| B. No Individualized treatment:   - Some medicines are trial and error- you take it for 6 weeks, and take it and let me know - Some people are allergic to certain medications - Everyone can’t take the same medication | 0 |
| C. Paying for the therapy is not easy   - So far as therapy, it does work, but you have constantly pay for that too - If something has to be continuously done, that doesn’t fit in everyday life, then - Most therapist will give you a lay pout of exercises that you can do, you can do them at home - If you are working every day, then you can’t do that - If you need therapy every 6-8 hours, you can’t do them while at work - May not be able to do exercises while at work - At home PT, it has to be included in your routine- taking 15 1minutes to do stretching, either before work; even while lying in bed, you can stretch your knee - You just have to figure the ways to ease their pain | 1 |
| G. Weight loss may have some effect on pain, but pain is still there   - helps keep the inflammation down in your knees and helps with blood pressure - May still have pain - May be different for individuals- some might help the pain, some it might not - May make the pain less severe but may not eliminate it. - Weight loss: definitely big, everyone can lose weight to keep down the pain: It does work - If it doesn’t work, someone is not mindful of their weight, you need to get rid of it for you and your knees - Either there is more damage to your knee than you think - One of the biggest things for osteoarthritis is weight loss - If your job is physical, weight loss can help a lot - I don’t know how it helps others, but all those help me - Health problems- thyroid problems, where you can’t lose weight; I thought everyone can lose weight - Some arthritis medications can make them gain weight, like steroids; some pills make them gain weight. - Therapy, exercise, weight loss helps with the pain - Weight loss, it decreases the pressure on your knees | 1 |
| E. Once the cartilage is gone, it’s bone to bone, nothing works then   - Wear and tear, comes from having to work constantly and use those joints - Your cartilage and your bones wear over time - when they are bending it, it is going to cause bone on bone effect. | 9 |
| F. The strength training differs from individual to individual: No individualization of the strength training   - different types of exercise that you can do to rebuild the strength in your knees - Physical therapy and massage therapy to help with the muscle strength - Some people can not do the exercises due to other things, medicine | 0 |
| H. Natural medicines, I believe, help   - I believe vitamin D from Sun can also help - It eliminates the prescription medicine - Some people may not like the regular medication - If it’s not too bad, they all work; if they don’t work, you need something else beyond that | 0 |
| I. As far as therapy, it’s an awesome idea, and then it’s long enough, they need to make it longer so that people can do exercises at home   - That sums it up, because it’s not long enough- you are not getting the help for 6 months; insurance is giving you only 6 weeks - Individual has to they are training you during the 6 weeks how to do therapy on your own– you need a routine each - Do it before and after the PT sessions - If you are benefiting from that, then you the individual has to do it at home - A lot of people can not do it on their own- they need the extra help. | 0 |
| J. Strain of physical work is interfering with treatment effectiveness:   - As you get up in age, your body changes and that’s not something you can change - Why do so many people get it? - A lot of people are on their feet, 6–8-hour shifts, the physical thing of walking up and down during work - Because you are damaging the nerve, nothing works - Employee who is on their feet 6-8 hours a day, lifting heavy weights, can put a strain on their knees. - UPS driver, stepping up and down, picking up a package, can flare up the pain | 8 |
| K. You like the ways to reduce the inflammation using the hot and cold pack to reduce swelling   - Additional things like hot and cold packs can improve the effectiveness of these interventions - I never tried ice packs; heat does better with pain | 0 |
|  |  |
| **NGT 10: 2 people; 2 M; 3 White (12 votes):** | |
| **A.** 3+ No cartilage left in my knees (and hips)   - Well, the reason I am doing the replacement is because I have no cartilage left in my knees and hips - The reason treatments don’t work, is that - I had groin pain with hips; knees and hips pop so bad, that’s why I use the walker, I knew I was going to fall | 3 |
| **B.** Prescribed Medicines don’t work I don’t think the medicines worked and did what they were supposed to do   - I REALLY don’t know - Sometimes I felt like guinea pig, when they tried different things on me. - I just don’t know why they didn’t work - I really haven’t tried any medications other then HCQ and walking and the exercise - Medicine hasn’t been changed, since I let him know about it, for the last months only | 0 |
| **C.** 1+ The pain; I had trouble with therapy and exercise with the pain   - I would say I am exercising now for my hip and it is helping me now. - At one time, I was learning to walk again; Back in 16-17, when I was in the hospital, I learnt how to walk, that’s when I ended up in PT - Work yourself through the pain; it was hard to do at first - I remember one time I was at SPAIN rehab in BHM; the therapist I had was making me do certain things, she was a mean woman, was making me mad - When I was learning how to walk, I had a male doctor, he made me mad also, he got me to walk - With my feet problem and knee problem, 24 on the treadmill makes it painful to do, before you get through the set time; I just do it through the pain - I don’t know why it’s getting difficult the more I do it. - I did have therapy a couple of years ago, they pushed me a little bit too hard; at times I think they hurt me more than they helped me. - Therapy helped me after my hip surgery, not sure outside of it, it helped. - I have started exercising and I have been walking 5 days a week, I think it’s pretty fast at 24 for me. | 1 |
| **D.** I wasn’t making any progress after some time with Therapy, I thought I could make more progress myself   - That’s the way I looked at it - I made a lot of progress myself; now I think I am ready to add therapy on top of my exercises. - I do 8 sets of 12 lifts with 10 lb dumbbells for many exercises | 0 |
| **E.** 3+2+ Weight loss is difficult to get and to maintain   - I also started dieting 1st few months, lost 20 lbs., and am stuck, haven’t lost any more weight - Nothing for breakfast other than a protein bar; lunch meat; dinner, cereal and something light - I have been working with a lot of weights for 30 minutes for the last couple of week. - When they reconstructed my abdominal wall in the hospital; I tried Atkins diet, protein went down from 250 to 208 lbs. - I barely eat anything, still can’t lose weight - My metabolism is slow; I have been big my whole life - I completely quit eating, may be that’s the wrong way to do it - Not knowing how to achieve it - I have always been big and it’s hard to keep my weight down | 5 |
| **F.** 2+1 Exercise may not work sometimes – a lot of people can’t do what I do- Motivation issues   - Exercise should work. It should work. I still work. I also walk 5 days a week. - Before my surgery, I did exercise, but I was not getting anywhere, I went to the back surgeon, he told me I had bulging discs, but it was my hip- I got the hip replaced and groin pain was gone. | 3 |
| **G.** Taking better care of yourself and your life. | 0 |
|  |  |
| **NGT 11: 2 people; 1 M 1 F; 2 White (12 votes)** | |
| **A.** Weight loss: Its daunting if you struggled with weight through your life, it could seem unachievable.   - Its tied to mental health status, most have been overweight for years. - The failure is really hard to overcome - Every time you do this | 0 |
| **B.** Weight loss: Lack of time to dedicate to weight loss   - I think all of the things we have talked about so far; they just take up time. It’s not just the time it takes you do that. It’s the time it takes you to process, what that looks like and how you go about it. The devil is in the details, it’s always in the details. - Weight loss: Lack of monetary resources - Fruits and fresh vegetables can be expensive and require thrifty shopping. Coupon-clipping, I don’t have time for that- makes it more difficult. | 0 |
| **C.** Weight loss: Food prep   - As far as food goes, makes nutritious food and be financially can be difficult, you have to make it from scratch; pre-prepped foods aren’t accessible to a lot of people; prep options are really important | 0 |
| **D.** Weight loss: Mental health aspects, depression and body image issues   - I think there is a huge connection between a person’s mental health status and their ability to actually effect change in their own life. depression and body image issues can make it very difficult to achieve weight loss. - Make it difficult To combat hopelessness you face - Have to have a friend or confidant when you attempt to lose weight, do the things you want to do. Someone you trust and won’t judge but will encourage you. - Always have to buy into it. | 0 |
| **E.** Confusion over contradicting diet plans   - There is a lot of information available about different ways you can lose weight. It’s very overwhelming even for someone working on a PhD. And I know how to look for primary literature – there is an obscene amount of information available for this. - There is a question around exercise and diet, but you have to have that plan I talk about. And people are not following it. From a personal experience, it took years for me to realize what I needed to do for my arthritis. | 0 |
| **F.** There is short term FU through medicine, and there is lack of modification effort- what was originally recommended doesn’t necessarily work   - I am thinking of limited access to long-term nutritional or diet plans for lifestyle modification. - I just think if you are having trouble, with whatever you originally set up with the nutritionist, it is difficult to follow-up with them and get additional recommendations. | 0 |
| **G. 3+3 Cost/Access**   - Therapy: There is lack of access to therapy due to the cost, with or without insurance - Weight loss: Lack of monetary resources - Medicines: Cost of medication | 6 |
| **H.** Exercise: this comes down to time availability   - I would say someone who is over-whelmed today won’t be able to take the time. It would be difficult for anyone. - I think there is a gold standard they want to maintain on a daily basis or 5 of the 7 days, and it can be hard, where on your schedule you can make time for those things. If you are juggling multiple things, it can be overwhelming. - It’s a matter for positive reinforcement – 45 minutes of cardio and then weight, it’s a lot of time, you just don’t’ see a lot changes, it’s disheartening. - There is a need for more arthritis docs; you spend an awful lot of time in the waiting room | 2 |
| **I.** Exercise: Pain especially and fatigue   - No one really wants to move when they are hurting a lot, walk up steps, or something simple lie gardening; constant pain can just be exhausting. Definitely barriers to trying to exercise - I agree totally; dealing with pain, has consequences depending on who your doctor is and the drugs they put you on, or give you can be very positive or very negative. Think that has a big impact on desire to do this. - I think people are so over-whelmed today with all this going on, and the issues facing our country, it would make it hard for a lot of people to even exercise or anything. - I believe that exercise doesn’t work, if you try to eat an elephant with one bite | 0 |
| **J.** Exercise: COVID, restricted access to weight training and other work-out facilities   - COVID has impacted people psyche, eye-opening - Masks are wonderful to help to decrease COVID transmission, they are required to go into exercise facility, makes it really difficult to go to. - Cost can be a barrier - A lot of people use the Gym, they get a little laxed in wiping things down, it’s just not safe. - Low impact exercises at the Gym are really well-suited - Exercise requirements of knee replacement were strenuous; I am fortunate that I have a cul-de-sacs, I can do laps in the neighborhood | 0 |
| **K.** Exercise: Lack of motivation   - I struggle with it a lot; I have a lot of demands and stressed because I am a grad student, have to walk at night just to get the exercise - Everybody is busy - You have a lot on your plate - Having a friend, you can talk to can help. - From my experience, I did not get serious about walking and things like that. I just think it’s hard, very hard. You just have to find stuff every day, that will motivate you, even if it’s a board, you check | 0 |
| **L.** Exercise: Psychology, you have tired it before and sometimes you don’t know how to change your behavior.   - I think some of the hardest to change are really behavior, and that in order to have treatments, like exercise to be effective, you have to be able to follow through – one of the most important aspects, - There are a lot of things that can get in the way - Some of the frustration, and feeling helpless can be big issue, or feeling overwhelmed - I agree; I think that you have to have a plan, and you have to stick to that plan; and it should not be a lot of things when you start - You should start simple and small - You should have support - Support if where I see some of the holes, and a lot is tied to health insurance. - Personal trainers are not available - People with arthritis are left out in cold, you get recommendations into home therapy; if you go into gym, and want to do anything serious, you have no idea; no way to tailor it to yourself - How to train core and lean muscles. - Got to have goals and start small | 4 |
|  |  |
| **NGT 12: 2 people; 2 F; 1 White 1 African American (12 votes)** | |
| **A.** Medicine: can be addicting, and unable to take for long periods   - When I have more pain, would usually take hydrocodone, then worried about addiction, it does help the pain quite a bit, and allows the better quality of life. - Sometimes it’s the easy way out - I am a retired nurse, so I know how addicting they are. So, I try to stay away from them. | 2 |
| **B.** Medicines are usually only usually providing temporary relief of the pain   - I use bio freeze, heating/ice packs – temporary - Topical creams are also short-term | 2 |
| **C.** Motivation for Weight loss and Exercise is very hard to do because of what you need to do exercising, due to difficulty in exercising due to the pain   - I see that benefit is obvious if you lose weight, not as much weight for the knees to carry around, but it’s more painful. You are already in pain; you add more to the pain. - May benefit someone who did not have advanced disease, or someone who is younger - I definitely know that weight loss will help. My doc told me 10 lb Weight loss will help. I have tried to cut down my sugars. Weight loss will help my other conditions. “My grandson said I need discipline”. - Some days you just get hopeless and you don’t’ have the motivation. | 3 |
| **D.** Physical Therapy: strengthens muscles but doesn’t help with pain   - I took physical therapy it didn’t help me at all - I did 14 visits to PT, and we were both in agreement there wasn’t a huge improvement in those visits - I have to use a cane, it’s embarrassing – is it something I have to live with - I went in expecting more and thinking that I will get more out of it. If it strengthens muscles, I expect it to help with the pain, and help me to exercise me more. | 0 |
| **E.** Injections: Short-term and painful   - For me, you go to have injections, have immediate relief for 3-months and it comes right back - Sometimes the pain after the injections - It’s temporary relief – it is short-term - I agree with all of it - I just had gel injections only 2 weeks ago, no huge difference with it, and no | 0 |
| **F.** Side effects of medicines   - Concern about side effects: What is all this medicine doing to our bodies, we are taking all these medicines. What side effects is it going to cause? - Due to my kidney insufficiency, can only take Tylenol, can not take NSAIDs. Bound to Tylenol and Tylenol only. Only take 1-2 Tylenol a day. - Due to my Fabry’s disease, avoiding meds that can affect my kidneys - *Side effects of steroids – gained more weight due to steroids due to my rheumatoid arthritis; haven’t been able to come off my prednisone for 20 years* | 1 |
| **G.** Exercise: Motivation due pain in movement   - Makes you gain weight - The last thing you want to do is walk or take exercise class, when you are in pain; some of it is my diet and my age - I will end up with pain if I do that - Affects you emotionally, people don’t see the pain you are suffering from in your knee3 - Interferes with exercise and PT. | 1 |
| **H.** Weight loss: challenges due to other medications   - steroids cause you to gain weight and you don’t feel like moving - I take a lot of medicines for my Fabry’s restless legs and some of those meds make you gain weight | 3 |
| **I.** Most of these are short-term solutions   - Never been told there is a definite cure, or a solution - Most things we do are short-term – constantly doing something to take care of the next 2-hours - All treatments are short-term - It feels like you are a hamster on a wheel, you are going over and over. You get up in the am, you rub down knees, bio freeze doesn’t work, then looking for relief with something else - Painless day is very rare - Would like to do a lot of things with grand-daughter and I have to say “nana can’t do these things, nana is hurting” | 0 |
| **NGT 13: 2 people; 1 M 1 F; 2 White (12 votes)** | |
| **A.** Medicine: help a little bit, but the problem is that it doesn’t happen quickly enough, the different things I take.   - I just started a new treatment, Forte, I do every day, is supposed to help bones not be as brittle - I also take MTX injections on the weekend and Enbrel shot on the weekends- on them for a while, I don’t feel like they are doing much but know what will happen if I quit taking; they cost a lot, and I am living on a disability check, I have to watch - I take Celebrex every day and I am maxed out on that; when I have stiffness, I can’t take anything other than Tylenol or Ultram, it was like water, it didn’t help, so I quit taking those - I take a Percocet 10 mg 4 times a day; but that’s going to change since I am going to a pain clinic next week, and that’s going to change maybe; I take HCQ twice a day; I do take a gabapentin 300 mg two of these three times/day – you would think with all of those I’d be dancing on the street, but I don’t know. - I had a knee replacement on my right knee, it doesn’t bother me near as much as the left knee; but I have stiffness in both knees: I have lupus and Sjogren’s. The left knee gives me the problem, and we can’t control the weather. - I take my medicine, when I first get up, sitting in the chair, I am ok. But If I stand up, the pain will shoot in my left knee. - It feels ok sitting here. Get up and it’s stiff. I have talked to people, and they have said that shouldn’t happen. | 6 |
| **B.** The weight loss part is a big one for me, but can’t do   - I was on prednisone for 2 years for lupus, I got to 240 lbs., have lost 50 lbs., but the left knee still bothers me. - I had lost weight last year, while this was going on, I lost 60 lbs., but I might feel good mentally, but it made no different to the knee- the pain and all that was still there. - I have fallen so many times, and maybe that’s why. I can’t get on my knees to get up, then I am laying on the floor, I had to call paramedics or my son-in-law to pick me up. I just lay there, letting my dog lick me. | 0 |
| **C.** I can’t do exercises because of the knees   - I have recently joined a Gym; I am the oldest one in my class. They do modify the exercises, so I can do them. It has helped me to feel a little better overall. But as far as helping with the stiffness or pain, I have not noticed the difference. - I belong to the Y; my wife and I used to go there twice a week. I felt better sitting in the Sauna, I 0would get out and swim in the pool and feel like a new man. By the time, I got back home, you feel like you haven’t done anything. - When I was taking therapy with stationary bikes, I would be ok for 2-3 minutes, I guess. You stayed on it for 8-minutes, it felt ok, but then when I stood up, it felt loose, nothing sturdy. The exercise works well while you are doing it, but when you quit it it’s a big difference. | 0 |
| **D.** Talking therapy: pool, water therapy is good, but now, they have been shut down due to the COVID   - Then COVID, shut down the Y. - With my immune system being the way it is, I can’t take a chance. You hear horror stories about it | 0 |
| **E.** Lack of Motivation for exercise:   - The other day, when I was exercising, my coach is little rough on me sometimes, she wanted me to do some push-ups. I told her I couldn’t do it. She asked me to get on the floor and do it. It wasn’t pretty, but I did it. I am scared of falling down and not being able to get up. I wouldn’t have it myself. - I have to have that motivation, someone telling me that I can do it. Because fear, it can grow, and overtake you. - That’s a true statement too. The fear is big. I live by myself; I think about that. If I fall down, who is going to pick me up and help me get up. - I have to walk all the way now at Kirklin clinic, and if I hadn’t had the special walker from my friend, I wouldn’t be able to do it without it. - If I had someone like Katrina’s coach, it would make me try. I would give it a shot. - It would be nice if the insurance would pay for it. I don’t understand why they wouldn’t cover it. Some companies give you a discount if you join a Gym. Why can’t insurance help with a coach | 4 |
| **F.** Brace, which I have works, but I need help to put it on, but I have nobody here to help me to put it on.   - I have a couple different ones, but the one that works really well, I can start to get it on, but I need to stand up, when putting it on, and I can’t do that. It does help. It makes me feel confident that knee won’t give out. - I also found when I was bigger on the prednisone and I have tried putting the brace on, it wouldn’t fit, and I had the extra-large, so I had to go without the brace for a while. - I went to the place, they measured me and Medicare payed for that with the metal in. It’s the one that fits me, my leg is swollen due to previous surgeries. | 0 |
| **G.** Creams are not strong enough for pain   - They are not strong enough, they are not. - Voltaren gel – it didn’t work. - I had diclofenac, my wife liked it on the hip, I put it on me, and it didn’t do nothing for me. It got expensive too. | 0 |
| **H.** Damage is done, even with weight loss and therapy   - Well, you know, with weight loss, you still have the damage, you get some pressure off, but pain-wise I couldn’t tell the difference. I am still working on losing weight, but I have lost 60 lbs. - When you are doing treatment, you are doing damage control. But the damage is there, so it’s still going to hurt. - I agree with what she said, even when I lost weight it was there - With my RA, the problem is there. They do not want to operate on the knee. | 2 |
| **I.** Pool are good, but they are expensive, and the insurance does not cover it.   - I had the silver sneakers card from Medicare, let’s me go to the Y for free, but - MY gym doesn’t offer the sauna and the pool. - I found a small pool at my Y - Insurance for me will only cover so many therapies for therapy. They said we will do pool therapy where I go for PT. It was like thousands, and I said sorry I can’t do it. | 0 |
|  |  |
| **NGT 14: 4 people; 2 F 2 M; 2 White, 2 African American (24 votes)** | |
| **A.** Pills and creams: Each individual is different, what might work for me, doesn’t work for another person   - I agree with that. - The application of creams, gels doesn’t seem too beneficial, for the amount of time and money you spend. - I found that pills that my doctor gave me, I didn’t like the feeling it put me in- relaxed feeling - Cream: it’s the way of doing it. Different types of creams have helped. - Cream that I got was the – caused me to have GI issues. Pre-existing conditions can be affected differently. - Prednisone makes you gain weight. - Everyone is different. Our physiology is different. | 6 |
| **B.** Once you feel better, you stop using the cream or stop using the pills. You don’t complete the treatment.   - That’s pretty true for me. - I don’t start using it until it starts aching again. Till it really starts hurting again. - For me, I have never reached this pivotal point, where I have had so much pain relief. | 0 |
| **C.** Not consistent, not taking the meds the same time every day.   - I have a child in school, I have a husband, I have a home. You put yourself in the last place, as a caregiver. - I am not at a place where I take medicine every day. I am not taking medicine every day, so this does not apply to me. - I take care of a physically challenged child, I need to make sure that I am doing everything for him that I need to do. - You have had this pain; you endure the pain every day. You work around it. You learn to live with it. - I don’t want to get dependent on the pills. | 0 |
| **D.** The weight loss: Don’t have the will power to keep going   - Once we have lost that 5 lb, that’s saying a lot. You say it’s ok, I have done that. You are not as disciplined to keep going. - I definitely agree, it’s exactly true. - I think trying to adjust your lifestyle to keep the weight from coming back on is a major problem. To stick to the regimen to eat the right food and get the right exercise daily weighs on you. Need to be disciplined to do that. - Pre-existing diabetes, heart failure, tend to psychologically prevent me from participating. Or at least giving me the will to participate continuously. - Weight loss: My doctor always tells me that if I lose some weight, it will help. If you lost weight, you don’t have that extra weight that your knee is trying to carry. | 3 |
| **E.** Become lazy with exercise and just give up   - You become complacent, with just not exercising - You know exercising will help, your schedule is so busy to put in the extra time to do exercise or go walking. My son is telling me let’s just walk up and down the street, by the time I am done taking care of him and cleaning the kitchen, you don’t have the time to put that exercise in your regimen. I even got a bicycle, yet to ride the bike for more than 2 minutes. Just not convenient, in your lifestyle. - Mine is always “I will do it tomorrow” and tomorrow never comes. I say I am going to start Monday; it never happens. | 0 |
| **F.** Weight loss and exercise: No instant gratification, as human beings a lot of times we don’t want to finish what we start if no gratification.   - If it’s not working, if I don’t see it right away. - You work out and work-out and nothing happens. - It goes along with your diet too, and you have made sacrifices, and you lost only 2 pounds. You are not seeing the benefits of all the sacrifices you are making just to lose those 2 pounds. - You put in on really easy, but you catch a devil to get it off, once you put it on. - The time restraint is a major consideration for me. Because I am a caregiver. To go to the Gym to go to the pool, those type of activities, I have time constraint. | 4 |
| **G.** Individual differences in disease: It strikes me as an important component of the individuals with pre-existing conditions, genetic predisposition to osteoarthritis.   - I think that’s true; everyone is different. | 4 |
| **H.** Pain with the physical therapy: Did not seem to be beneficial for the length of time, I did go. I didn’t seem to feel that I was benefiting much from it, other than the pain from the exercises. | 2 |
| ***I.*** *Problem taking medications due to other diseases: I found that most of the medications I tried had limitations. For example, taking the anti-inflammatory agents, because of my liver and kidney damage, they were not advised for long-term use.* | *0* |
| **J.** Difficulty in getting opioid Pain meds: Very difficult to get anything for actual pain, other than the anti-inflammatory agents | 3 |
| **K.** Medicine works temporarily, because I feel that it numbs the information in your brain to stop hurting. Puts you in melatonin state to not feel the aching and hurting. | 2 |
| ***L.*** *You don’t incorporate the therapy into lifestyle, once you stop the therapy.* | *0* |
|  |  |
